# Graphdiyne bearing pillar[5]arene-reduced Au nanoparticles for enhanced catalytic performance towards the reduction of 4-nitrophenol and methylene blue†

**DOI:** 10.1039/c9ra07347g

**Published:** 2019-11-25

**Authors:** Xiaoping Tan, Jianhua Xu, Ting Huang, Sheng Wang, Maojie Yuan, Genfu Zhao

**Affiliations:** Chongqing Key Laboratory of Inorganic Special Functional Materials, College of Chemistry and Chemical Engineering, Yangtze Normal University Fuling 408100 China xptan@yznu.cn fu1165158114@126.com

## Abstract

Graphdiyne (GD), a novel two dimensional (2D) carbon material, has earned a lot of attention in recent years. Constructing a novel hybrid nanomaterial based on GD, macrocyclic host and Au nanoparticles is an effective strategy for heterogeneous catalysis applications. While tremendous advancements in the preparation of two dimensional (2D) materials anchoring Au nanoparticles have been made, it is an urgent requirement to explore a green, efficient and facile approach for obtaining small-sized Au nanoparticles. The use of the 2D material graphdiyne (GD) presents more-promising candidates for constructing excellent sites for loading metal nanoparticles. In this study, a novel 2D heterogeneous hybrid nanomaterial (P5A-Au-GD) based on GD and pillar[5]arene (P5A)-reduced Au nanoparticles (P5A-Au) was successfully prepared. In this strategy, the P5A can reduce HAuCl_4_ with the aid of NaOH in the dispersion of GD. Accordingly, the generated P5A-Au can immediately interact with GD to form the P5A-Au-GD hybrid nanomaterial without any harsh reduced materials or other energies. The Au nanoparticles with average diameter of 2–3 nm are homogeneously dispersed on the surface of GD. The heterogeneous 2D catalyst of P5A-Au-GD shows high catalytic performances in the reduction of 4-nitrophenol and methylene blue by comparing commercial Pd/C catalyst. Meanwhile, the unique 2D heterogeneous hybrid material P5A-Au-GD exhibits durable recyclability and stability during the catalytic reaction. Considering the outstanding merits of the heterogeneous 2D catalyst of P5A-Au-GD as well as the simple and green preparation, this study might not only present enormous opportunities for the stabilized, high-performance and sustainable catalysts but also be applied in other frontier studies of sustainable functionalized nanocomposites and advanced materials.

## Abstract 2

Application of a novel 2D heterogeneous hybrid nanomaterial based on graphdiyne and pillar[5]arene-reduced Au nanoparticles in catalyzing the reduction of 4-nitrophenol and methylene blue.

## Graphical abstract

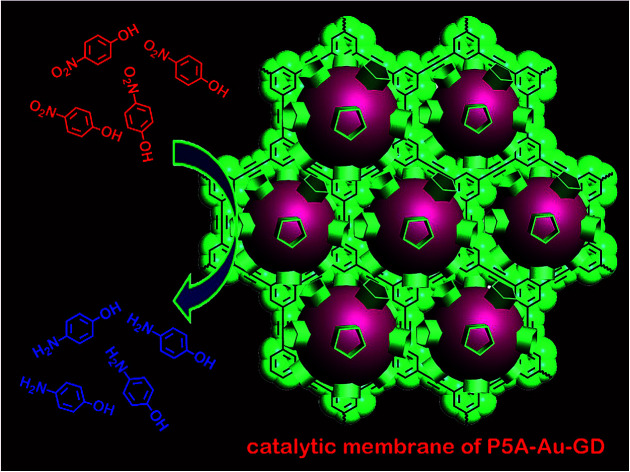

## 1. Introduction

Carbon materials, such as carbon nanotube, porous carbons and graphene, have received enormous attention owing to their potential technological value in sensing, catalysis, batteries, nanoelectronics and so forth.^1–3^ Among these carbon materials, the two dimensional (2D) carbon materials are frequently studied due to their excellent properties including optical electrical properties. The 2D polymers, constructed by covalent bond linkages are the novel 2D materials.^4,5^ Graphdiyne (GD), generated by sp- and sp^2^-hybridized 2D graphene-like material, shows highly conjugated π system, controllable electronic property and conformably distributed pores, and can be used in different fields comparable to conventional sp^2^-hybridized carbon materials.^6–11^ Owing to the unique properties of GD, it can be applied in solar cells, electronic devices, rechargeable batteries, biomedicine and therapy and advanced materials.^12–16^ Furthermore, GD is a protruding 2D material for anchoring metal nanoparticles, which is increasingly becoming a promising 2D material.^17–19^

With these outstanding features in mind, we study the integration of the flat sp- and sp^2^-hybridized GD and Au nanoparticles for catalytic applications.^20^ We anticipate that the interaction between Au nanoparticles and the alkyne and aryl π-conjugated networks of GD would be beneficial for stabilizing Au nanoparticles from aggregation.^21,22^ In addition, the preeminent porous structure of GD could embed the Au nanoparticles with a forceful adsorption energy.^23,24^ Finally, the splendid chemical stability and electrical conductivity allow GD to act as 2D material support for the catalysts. Nevertheless, the harsh reductants (including NaBH_4_, NH=NH and others) are applied during the procedure of generating Au nanoparticles, which is harmful to the environment and goes against sustainable development. Therefore, constructing a green and environment-friendly strategy to prepare Au nanoparticles and obtain sites on the surface of Au nanoparticles is more and more crucial and significant.

Macrocyclic host modified metal nanoparticles are an effective method in order to obtain small and functionalized nanoparticles.^25,26^ Macrocyclic molecules play a critical role as building blocks in supramolecular chemistry, especially serving as an important host in supramolecular host–guest recognition.^27,29^ Among diverse hosts, calixarenes, cyclodextrins, cucurbiturils and pillar[*n*]arenes are the most interesting due to their simple synthesis and great versatility towards chemical modification, presenting them with tailored recognition properties.^30–34^ The host mediated preparation of metal nanoparticles provides a good research procedure and promotes their performances and potential values.^28,35^ Interestingly, Zhao *et al.* reported a green and environment-friendly method for preparing Au nanoparticles based on redox reaction between hydroxylatopillar[5]arene and HAuCl_4_ at room temperature with the aid of NaOH.^36^ The obtained Au nanoparticles have a uniform size of ∼5.0 nm. This study offers an important avenue for synthesizing Au nanoparticles without employing harsh reducing agents. We preconceived that the size or catalytic activity of Au nanoparticles might exhibit great change by introducing the 2D material during the process of preparing Au nanoparticles as the obtained hybrid nanomaterial could show multiple glorious properties of Au nanoparticles and the 2D material.

**Scheme 1:**
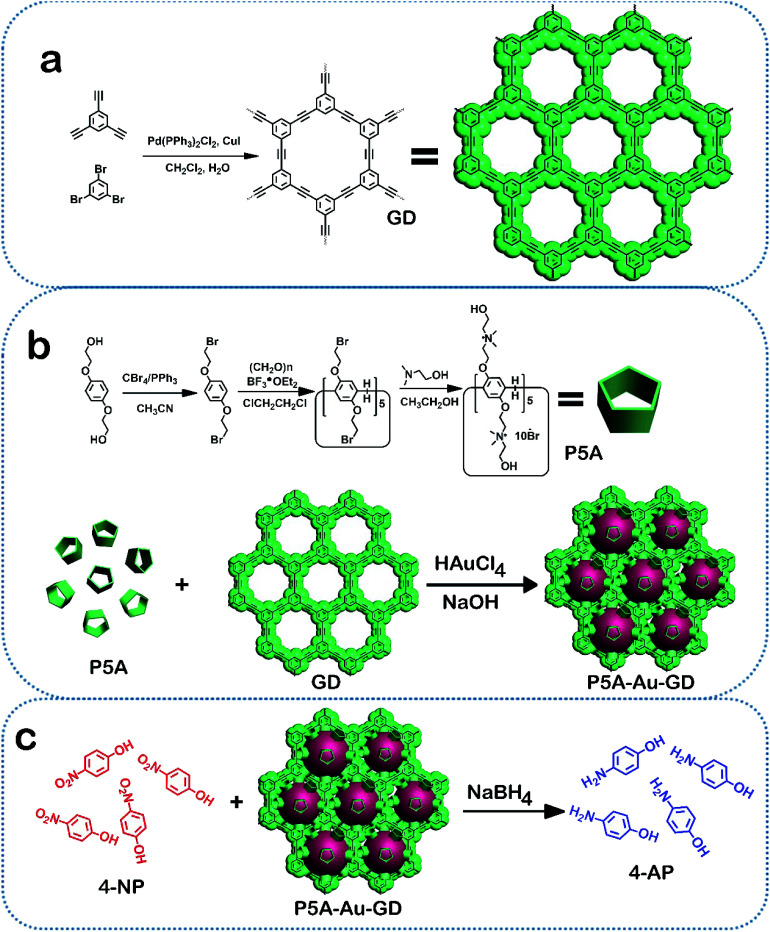
Synthesis of the P5A-Au-GD hybrid nanomaterial (a and b) and its catalytic application (c).

In this study, a novel hybrid nanomaterial based on GD, pillar[5]arene and Au nanoparticles was synthesized by a green approach. Firstly, the Au nanoparticles were facilely obtained by the redox reaction between hydroxyl pillar[5]arene (P5A) and HAuCl_4_ at room temperature without using any harsh reducing agent. This process of synthesizing Au nanoparticles was carried out in the presence of GD. This solution creates a coordination environment so that the formed Au nanoparticles can interact immediately with GD, which prevents the size growth of Au nanoparticles. Consequently, the pure 2D hybrid material of P5A-Au-GD with Au nanoparticles with a diameter of 2–3 nm can be obtained by centrifugation and washing with ultrapure water and ethanol. In order to study the catalytic activity of the P5A-Au-GD, 4-nitrophenol (4-NP) and methylene blue (MB) were reduced by NaBH_4_ in the presence of P5A-Au-GD. The heterogeneous 2D material of P5A-Au-GD exhibits high catalytic performances for the reduction of 4-NP and MB in comparison to P5A-Au and commercial Pd/C catalyst. Due to the insolubility of P5A-Au-GD in water, it can be repeatedly used without changing the morphology, size and catalytic activity of the Au nanoparticles. Considering the outstanding merits of P5A-Au-GD heterogeneous 2D catalyst as well as the simple and green preparation, this research might not only present enormous opportunities for the stabilized, high-performance and sustainable catalyst but also be applied in other frontier studies of sustainable functionalized nanocomposites and advanced materials (Scheme 1).

## 2. Experimental

### 2.1. Materials

HAuCl_4_, 4-NP, NaBH_4_, commercial Pd/C (10 wt% Pd) and methylene blue (MB) were purchased from Shanghai Titan Scientific Co. Ltd. P5A was obtained according to the published works.^37,38^ All aqueous solutions were prepared in ultrapure water. The apparatus and characterization are given in the ESI.†

### 2.2. Synthesis of GD

GD was prepared according to the method reported in the previous studies.^12,39^ 200 mg 1,3,5-triacetylenylbenzene was dissolved in 200 mL CH_2_Cl_2_ by stirring with a glass rod. Then, 150 mL deionized water (DW) was added slowly along the wall of the instrument, forming the aqueous phase of the two phases. Next, 150 mg Pd(PPh_3_)_2_Cl_2_, 30 mg CuI and 420 mg 1,3,5-tribromobenzene were added into 50 mL DW. The formed aqueous solution was dissolved as much as possible by a series of treatments such as stirring and ultrasonication. At last, the above solution was added dropwise slowly into the aqueous phase. The entire mixture was allowed to react for 72 h at room temperature. A continuous uniform large film was obtained, which was washed with DMF and acetone subsequently and a black film was obtained. The black film was dried under vacuum at 60 °C for 8 h in order to yield GD.

### 2.3. Preparation of P5A-Au-GD

0.5 mL 5.0 mM aqueous solution of HAuCl_4_ was added into 20.0 mL dispersion of synthetic GD (5.0 mg) and P5A (2.5 mg). The above mixture was continuously stirred for 15 min at room temperature. Subsequently, 0.2 mL fresh aqueous solution of 0.5 M NaOH was poured into the aqueous dispersion of GD, P5A and HAuCl_4_, and the mixture was stirred for 10 min at room temperature. Finally, the reaction mixture was centrifuged (16 000 rpm, 30 min) two times and the generated solid was collected and washed with DW and ethanol two times. The treated solid was dried *via* lyophilization under vacuum condition to obtain pure P5A-Au-GD nanocomposite. The P5A-Au was also obtained according to the reported work.^36^

### 2.4. Catalytic activity of P5A-Au-GD towards the reduction of 4-NP and MB

The process of catalytic reduction of 4-NP was carried out as follows: a 4.0 mL standard cuvette was charged by mixing an aqueous solution of 4-NP (0.4 M, 2.0 mL) and NaBH_4_ (0.5 M, 1.0 mL). Then, a dispersed aqueous solution of P5A-Au-GD in DW (1.0 mg mL^−1^, 10.0 μL) was added into the mixture of 4-NP and NaBH_4_. The reduction procedure was monitored at different times by UV-vis spectroscopy with wavelength varying from 600–200 nm. The controlled experiments were carried out under the same conditions as P5A-Au-GD. The only difference is that P5A-Au-GD is replaced by Pd/C.

The degradation of MB was also carried out by the P5A-Au-GD nanomaterial in the presence of NaBH_4_. To a solution of MB (200.0 μM, 2.0 mL) in DW, NaBH_4_ (0.5 M, 1.5 mL) was added under room temperature. Then, the P5A-Au-GD (10.0 μg) catalyst was added and UV adsorption spectrum was recorded in the range of 800–400 nm. The control experiments of Pd/C catalyst were studied in a similar process and the only difference was that P5A-Au-GD was replaced by Pd/C.

## 3. Results and discussion

### 3.1. Characterization of P5A-Au-GD

**Fig. 1:**
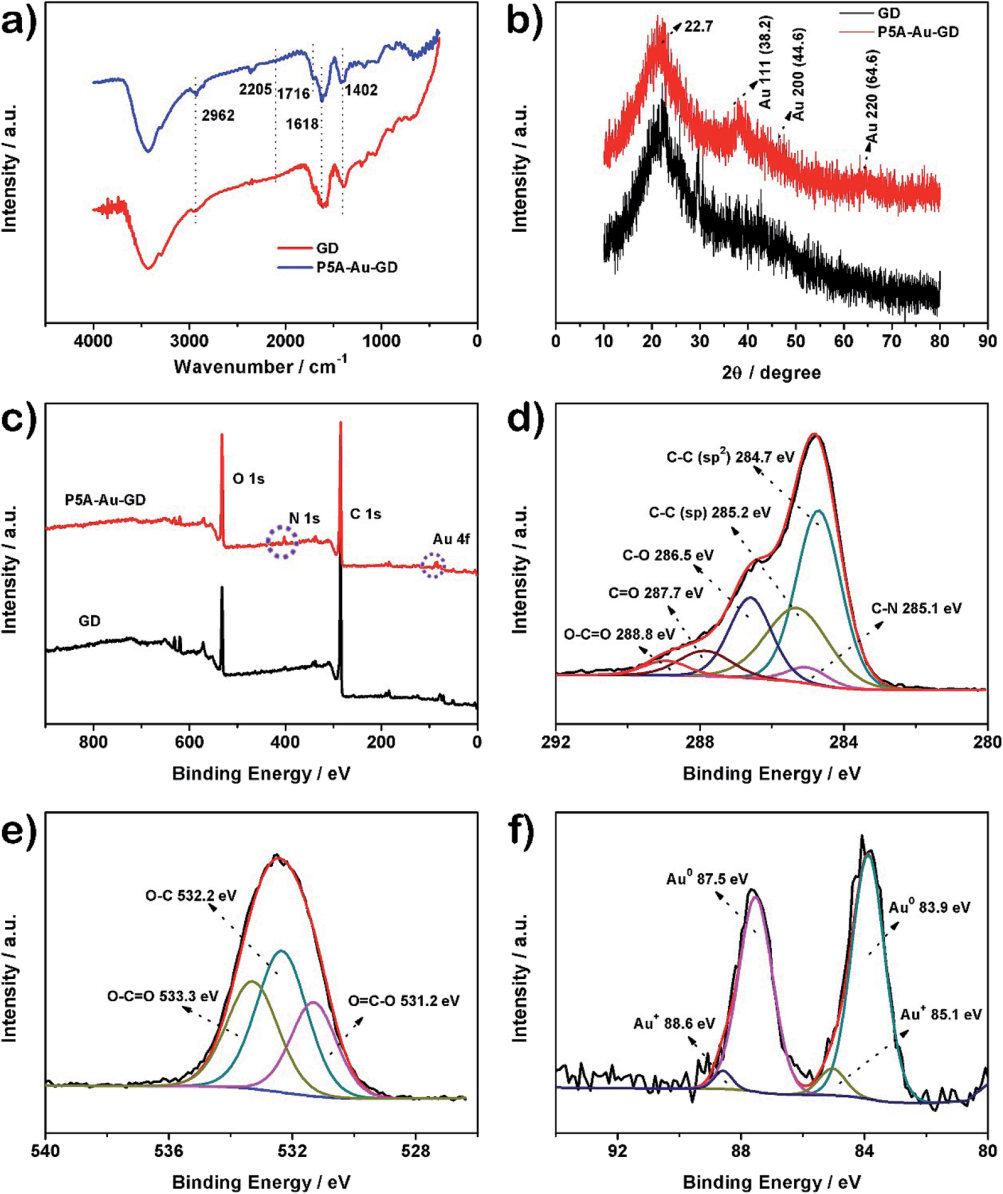
The characterization of the as-prepared GD and P5A-Au-GD. (a) FTIR spectra; (b) XRD patterns of GD and P5A-Au-GD; (c) XPS survey of GD and P5A-Au-GD; high resolution XPS spectrum of (d) C 1s, (e) O 1s and (f) Au 4f for P5A-Au-GD.

The prepared materials of GD and P5A-Au-GD were characterized by Fourier transform infrared (FTIR) spectroscopy. As shown in Fig. 1a, for the FTIR spectrum of GD, the bands at 2962 cm^−1^ and 2205 cm^−1^ are ascribed to the C–H and C≡C bonds, respectively.^12,39,40^ The FTIR spectrum of P5A-Au-GD displays the O–C=O peak at 1716 cm^−1^. These peaks can certify that GD is functionalized by P5A-Au. The Raman spectroscopy had been carefully carried out for the characterization of GD film. Raman spectrum can further certify the presence of sp^2^ carbon and acetylene bonds in the structure of GD (Fig. S1†). Crystal structures of GD and P5A-Au-GD were evaluated by powder X-ray diffraction (PXRD). As shown in Fig. 1b, the peak at 22.7° for GD corresponds to the interlayer spacing of 3.93 Å according to the reported literature.^39^ Meanwhile, the typical peaks of Au nanoparticles can be clearly observed and the peak at 22.7° of GD has not changed during the anchoring of Au nanoparticles. The result can prove that the Au nanoparticles were successfully synthesized and loaded onto the surface of GD and the framework of GD was not destroyed by the loading of Au nanoparticles.

X-ray photoelectron spectroscopy (XPS) is a credible technique for further assessing the incorporation of Au nanoparticles within GD. Fig. 1c shows that O, N and Au elements are detected in the XPS spectrum of P5A-Au-GD, which further suggests that GD is modified by P5A reduced and stabilized Au nanoparticles. Comparative high resolution XPS (HRXPS) spectra of C 1s for P5A-Au-GD (Fig. 1d) and GD (Fig. S2†) was recorded. The HRXPS spectrum for P5A-Au-GD exhibits the C–C (sp^2^), C–C (sp), C–N, C–O, C=O and O–C=O peaks, which is in good agreement with the structure of GD and P5A-Au. The HRXPS of O 1s for P5A-Au-GD nanomaterial (Fig. 1e) shows three peaks at 531.2, 532.2 and 533.3 eV, generating from the oxidized groups of P5A in P5A-Au-GD. These peaks are caused by the oxidation of hydroxyl groups in comparison with the initial P5A (Fig. S3 and S4†). The HRXPS spectrum of N 1s for P5A-Au-GD (Fig. S5†) reveals one distinct peak at 402.2 eV, which is associated with the secondary nitrogen of P5A. Finally, the HRXPS of Au nanoparticles (Fig. 1f) displays peaks at 83.9, 85.1, 87.5 and 88.6 eV, which are assigned to Au^0^ and Au^+^, respectively. According to the published works, the mechanism of P5A reduced and modified Au nanoparticles had been researched in detail. NaOH has only acted as a catalyst and the concentration of NaOH does not affect the size and morphology of Au nanoparticles. The loading of gold was carefully assessed by XPS and ICP, finally revealing a relatively average Au content of 3.67–4.02 wt% (Table S1†).

### 3.2. Microstructure characterization of P5A-Au-GD

**Fig. 2:**
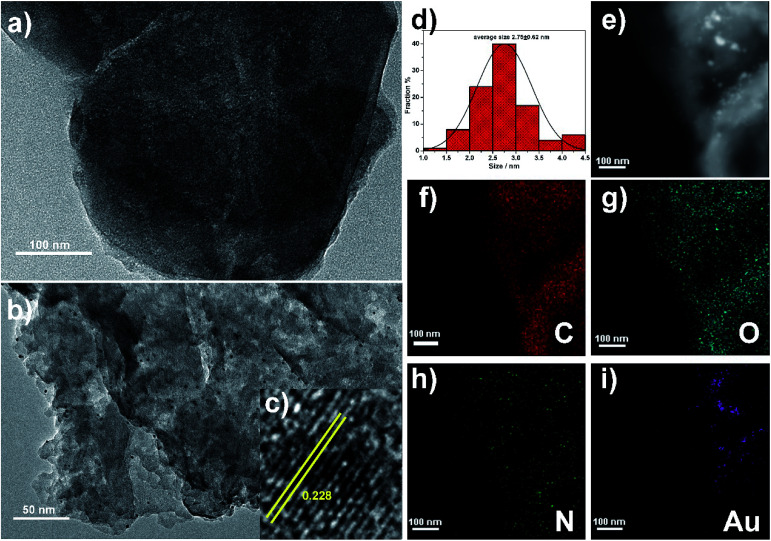
(a) TEM image of GD and (b) P5A-Au-GD; (c) HRTEM image of P5A-Au-GD; (d) size distribution of Au nanoparticles; (e) HRTEM mapping images of P5A-Au-GD: mixed element map; (f) C, (g) O, (h) N and (i) Au.

The microstructure of P5A-Au-GD was also studied using transmission electron microscopy (TEM). First, the TEM image of GD (Fig. 2a) exhibits the 2D nano-sheet morphology. Then, the Au nanoparticles were uniformly loaded onto the surface of GD (Fig. 2b). High resolution TEM (HRTEM) image of P5A-Au-GD demonstrates the highlighted crystal lattice spacing with 0.228 nm Au nanoparticles (Fig. 2c) and the size of Au nanoparticles is measured as shown in Fig. 2d, which implies that the diameter of Au nanoparticles is approximately 2.75 ± 0.62 nm. For comparing the size of Au nanoparticles with 5.86 ± 0.65 nm of P5A-Au, the size of P5A-Au is monitored as shown in Fig. S6 and S7.† Small size Au nanoparticles can be obtained in the P5A-Au-GD hybrid nanomaterial, which is attributed to the excellent anchoring effects of GD. More importantly, the morphology of GD has not changed after anchoring the Au nanoparticles, suggesting that the properties of GD have not been destroyed. Additionally, the elemental mapping (Fig. 2e–i) of P5A-Au-GD can further illustrate the distribution of C, N, O and Au on the surface of the synthesized 2D nanocomposite.

### 3.3. Catalytic activity of P5A-Au-GD

**Fig. 3:**
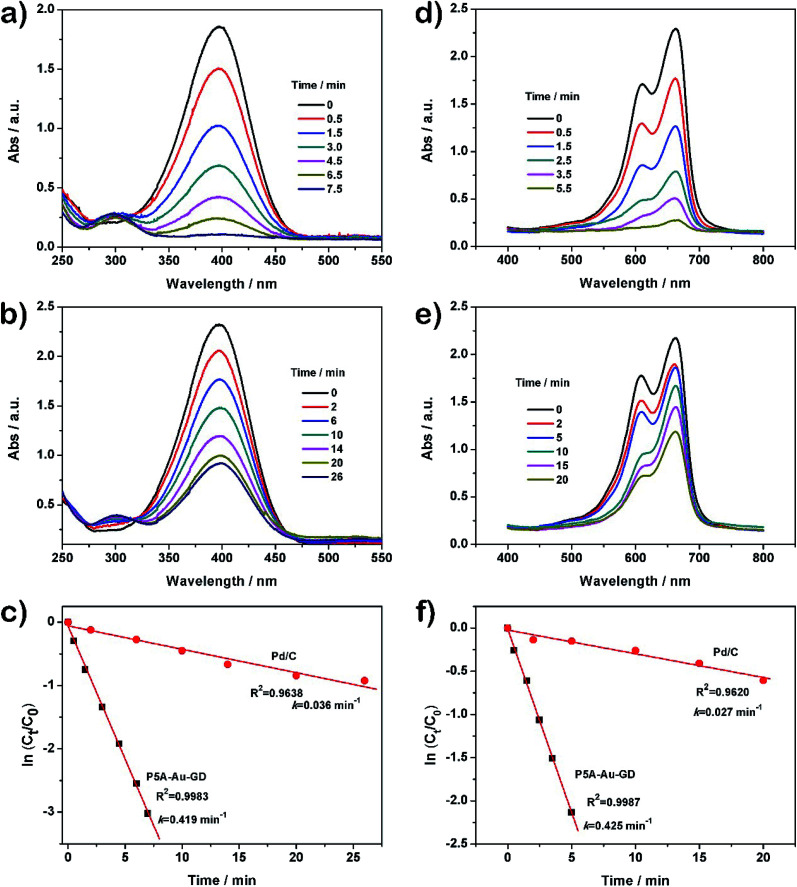
Time-dependent UV-vis absorption spectra for the catalytic reduction of 4-NP in the presence of (a) P5-Au-GD and (b) Pd/C catalysts; (c) plots of ln[*C*_*t*_/*C*_0_] as a function of reaction time for the reduction of 4-NP; (d) time-dependent UV-vis absorption spectra for the catalytic degradation of MB in the presence of P5-Au-GD and (e) Pd/C catalysts; (f) plots of ln[*C*_*t*_/*C*_0_] as function of reaction time for the degradation of MB.

The nitro group on nitrobenzene can be reduced by NaBH_4_, but the rate of the reaction progress is very slow. However, the efficiency can be enhanced obviously by using the P5A-Au-GD hybrid nanomaterial. As a result, the scheme of catalyzing the reduction of 4-NP by the prepared nanocomposite is frequently applied for studying the catalytic performance of the catalyst.^17,40–43^ According to these studies, the catalytic activity of P5A-Au-GD is investigated by catalyzing the reduction of 4-NP in the presence of NaBH_4_. As demonstrated in Fig. S8b,† the color of the aqueous solution of 4-NP is light yellow. After the addition of NaBH_4_ solution, the color of the mixed solution becomes bright yellow. Concurrently, the UV-vis absorption peak at the wavelength of ∼317 nm (Fig. S8a,† black curve) disappears and a new peak at ∼317 nm (Fig. S8a,† red curve) appears in the UV-vis absorption spectra after the addition of aqueous solution of NaBH_4_. These results suggest that the formation of the 4-nitrophenolate ion is in good agreement with the reported work. Moreover, the peak intensity of 4-NP did not reduce after 10 min of addition of NaBH_4_ (Fig. S8a,† blue curve). However, the peak intensity at ∼400 nm decreases dramatically after the addition of the dispersed solution of P5A-Au-GD (Fig. 3a). A fresh peak at the wavelength of ∼310 (Fig. 3a) appears, revealing that 4-NP is reduced into 4-aminophenol (4-AP) according to the previous studies. After 7.5 min, the peak at ∼400 nm disappears completely, implying that 4-NP is reduced to 4-AP entirely. This reaction procedure can be reflected in the UV-vis absorption spectrum, in which the peak at ∼400 nm is gradually decreased and the peak at ∼310 nm is increasingly enhanced. Meanwhile, the mixed aqueous solution becomes colorless (Fig. S9b†).

Commercial Pd/C catalyst was studied to compare the catalytic activity of P5A-Au-GD. With a similar process, although the peak at ∼400 nm can be decreased significantly, it needs more time than the P5A-Au-GD catalyst (Fig. 3b), displaying the poor catalytic performance of Pd/C than the P5A-Au-GD catalyst. For clear comparison of the catalytic performance, the rate constant *k* is introduced. In this system, the UV-vis absorbance intensity is proportional to the concentration of 4-NP. Thus, the value of ln(*A*_*t*_/*A*_0_) is equal to the value of ln(*C*_*t*_/*C*_0_). The *C*_*t*_ and *C*_0_ reflect the amounts of 4-NP at time *t* and 0, respectively. Therefore, the rate constant *k* can be obtained according to the following equation: ln(*C*_*t*_/*C*_0_) = *kt*.^36^ The time-dependent UV-vis absorption spectra of 4-NP reduction catalyzed by P5A-Au-GD and Pd/C is displayed in Fig. 3c. As a result, the *k* value was calculated to be 0.419 and 0.036 min^−1^ according to the slope of the fitted line for P5A-Au-GD and Pd/C, respectively. The *k* value for catalyzing the reduction of 4-NP by P5A-Au-GD nanocomposite is 11.3-fold higher than that of the commercial Pd/C catalyst. By comparing the *k* value, it is clear that the P5A-Au-GD nanomaterial exhibits superior catalytic activity than Pd/C.

The catalytic performance of P5A-Au-GD is further studied by catalyzing the degradation of MB. The pure MB solution shows a typical blue color and no visible color change is seen by the addition of NaBH_4_ (Fig. S9c†). The bright blue color undergoes no visible change after the addition of P5A-Au-GD (Fig. S9d†). Additionally, the catalytic degradation procedure of MB by P5A-Au-GD is monitored accurately *via* the time-dependent adsorption spectra at various times regularly. As shown in Fig. 3d, two characterized peaks were observed at ∼613 and ∼664 nm, which can be ascribed to the dimer and monomer of MB, respectively.^41,44^ Moreover, the intensity of these peaks decreases gradually until it disappears completely within 5.5 min after the addition of P5A-Au-GD, suggesting the ultrahigh catalytic performance of the P5A-Au-GD nanocomposite. For comparison, the catalytic performance of Pd/C under the same condition is also assessed for the degradation of MB (Fig. 3e). And the reactive rate *k* for the catalytic degradation of MB by P5A-Au-GD and Pd/C is calculated to be 0.426 and 0.027 min^−1^ according to the fitted line (Fig. 3f), respectively. The *k* value for the catalytic degradation of MB by P5A-Au-GD is 15.77 times higher than that of Pd/C, indicating the superior catalytic activity of the P5A-Au-GD catalyst.

In the reduced system of 4-NP and MB, the P5A-Au-GD shows superior catalytic performance, which can be ascribed to the synergistic effects between Au nanoparticles and 2D GD. The small sized Au nanoparticles can be obtained by the excellent 2D material GD, which can present a good deal of coordination environment for anchoring the metal nanoparticles.^20,45–47^ Owing to the excellent loading sites of GD, Au nanoparticles with uniform and small diameters can be obtained. On the one hand, the O and N elements in P5A have innumerable lone pairs of electrons, which causes the surface of the obtained Au nanoparticles to emit plenty of electrons. On the other hand, 4-NP exhibits an electron-deficient behavior due to the presence of nitro group, which is a strong electron-withdrawing group. Consequently, 4-NP can easily interact with the electron-rich Au nanoparticles. Furthermore, the extraordinary 2D and porous structure of GD is extremely useful to accumulate nitrophenol on the surface of GD (Fig. S10†). Definitely, more amount of nitrophenol can be adsorbed on the surface of GD due to the outstanding host–guest recognition between P5A and nitrophenol.^48,49^ These beneficial conditions can create higher chances of communication between Au nanoparticles and nitrophenol and MB.

**Fig. 4:**
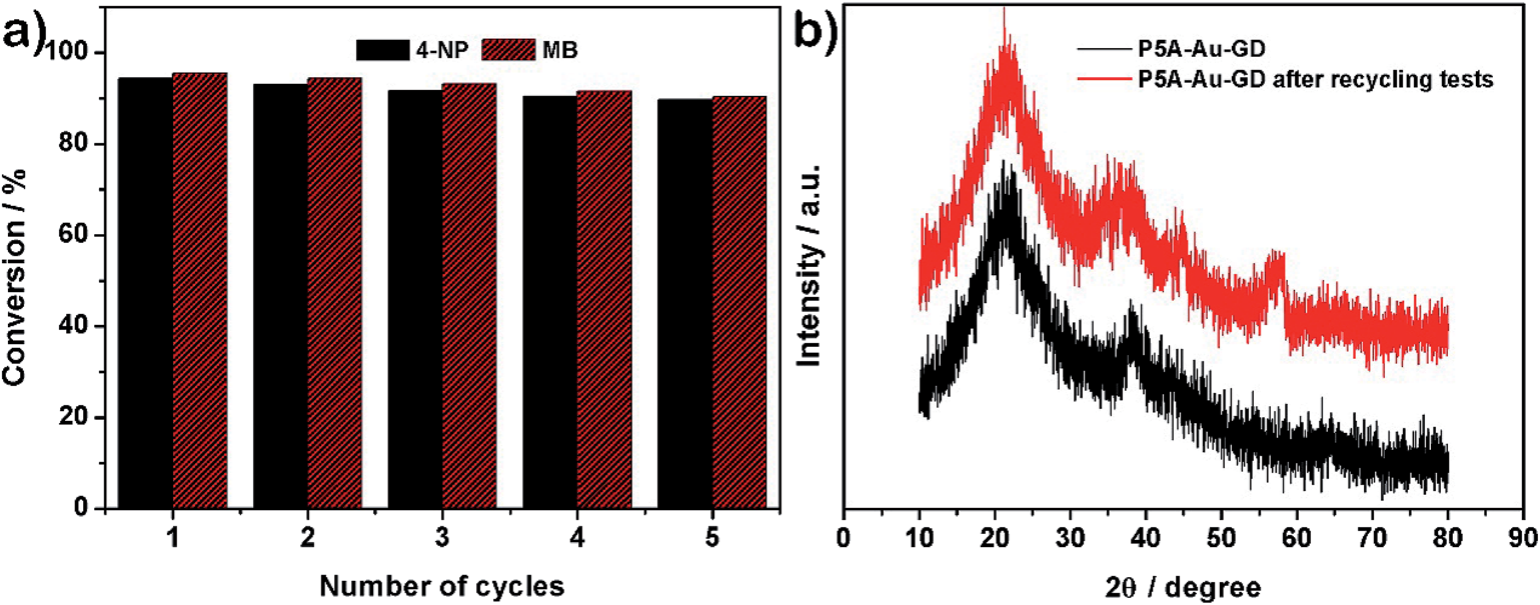
(a) Recyclable performance of P5A-Au-GD towards the reduction of 4-NP and MB over 5 cycles; (b) PXRD patterns of P5A-Au-GD before and after recycling tests.

**Fig. 5:**
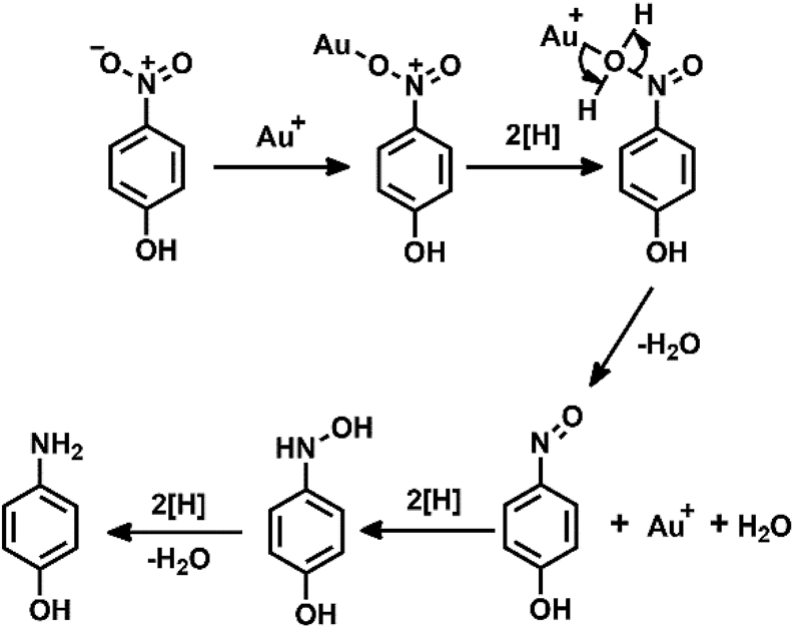
The proposed mechanism for the P5A-Au-GD catalyzed reduction of 4-NP.

Additionally, the recyclability and stability of the prepared P5A-Au-GD catalyst were also investigated. Fig. 4a illustrates the outstanding reusability without obvious reduction in conversion after five cycles. Furthermore, Fig. 4b exhibits the PXRD patterns of P5A-Au-GD before and after the catalytic reduction of nitrophenol, showing no obvious change after the catalytic reduction. Therefore, the unique 2D heterogeneous hybrid material P5A-Au-GD exhibits high catalytic activity and durable recyclability. As shown in Fig. S11,† there are no agminated Au nanoparticles, and the size of Au nanoparticles slightly decreases, implying high stability of P5A-Au-GD catalyst. This result is in good accordance with XRD analysis. According to the above analysis, the proposed reaction mechanism using P5A-Au-GD as the catalyst is displayed in Fig. 5, which is similar to the reported literature.^50^

## 4. Conclusions

In summary, a novel and heterogeneous nanomaterial based on the two dimensional material graphdiyne, supramolecular pillar[5]arene and Au nanoparticles was successfully prepared, which broadened the prospective applications of catalysis. Meanwhile, P5A-Au-GD derived from GD and Au nanoparticles was designed for the first time, which has great potential applications in advanced material preparation, catalytic reduction of nitrophenol and so forth. In general, Au nanoparticles are one of the most efficient metallic catalysts towards various reactions with remarkable yield and high turnover frequency. However, Au nanoparticles easily aggregate, which limits their further application. With its extraordinary durability and separability, GD is strongly considered to be capable of satisfying the urgent need of metallic nanoparticle supports. Furthermore, the Au nanoparticles were obtained by a clean and green approach, which has a significant value for sustainability. As expected, Au nanoparticles with GD confinement present desirable performance in controlling nucleation and seed growth of Au nanoparticles with small size distribution owing to the sufficient loading sites of GD. Particularly, owing to the insolubility of GD, the promising P5A-Au-GD nanomaterial exhibits recyclability and stability during the catalytic reduction reaction of 4-NP and MB. Accordingly, we anticipate that the 2D heterogeneous hybrid material P5A-Au-GD will have potential value in sustainable development.

## Conflicts of interest

There are no conflicts to declare.

## Supplementary Material

RA-009-C9RA07347G-s001
