# Supplementary material for: Graphdiyne bearing pillar[5]arene-reduced Au nanoparticles for enhanced catalytic performance towards the reduction of 4-nitrophenol and methylene blue
Source: RSC Adv. 2019 Nov 25;9(66):38372–80. doi: 10.1039/c9ra07347g (PMC9075914; doi:10.1039/c9ra07347g)
Supplement: RA-009-C9RA07347G-s001 [file RA-009-C9RA07347G-s001.pdf]

Electronic Supplementary Material (ESI) for RSC Advances.
This journal is © The Royal Society of Chemistry 2019

Supporting Information

Graphdiyne Bearing Pillar[5]arene-Reduced Au Nanoparticles for Enhanced Catalytic Performance towards Reduction of 4-Nitrophenol and Methylene Blue

Xiaoping Tan *, Jianhua Xu, Ting Huang, Sheng Wang, Maojie Yuan, Genfu Zhao *

Chongqing Key Laboratory of Inorganic Special Functional Materials, College of Chemistry and Chemical Engineering, Yangtze Normal University, Fuling 408100, China.

S1. Apparatus and Characterization: Hitachi Himac CR21G high speed refrigerated centrifuge (Hitachi, Japan) with 14000 rpm for 30 min was used to clean the synthetic sample. Ultraviolet−visible light (UV−vis) absorption spectra with wavelength of 200−800 nm by Hitachi U-2900 UV−vis spectrometer (Hitachi, Japan) was used to monitor the reduced procedure of *p*-nitrophenol and degradation of MB. The microstructure of the prepared sample of P5A-Au-GD was characterized by transmission electron microscopy (TEM) via JEM 2100 TEM instrument (JEOL, Japan) with an accelerating voltage of 200 kV. The crystal structures of GD and P5A-Au-GD are evaluated by powder X-ray diffractometry (PXRD) spectrum by Rigaku TTR III X-ray diffractometer (Rigaku, Japan) with a 2θ angle range from 10° to 80°. Fourier transform infrared (FTIR) spectra of synthetic materials was assessed on the Nicolet IS10 (Thermo Fisher Scientific, U.S.A.) using KBr pellets. X-ray photoelectron spectroscopy (XPS) was recorded by K-Alpha+ (Thermo Fisher Scientific, U.S.A.) where the source of radiation was mono Al Kα. NMR spectra were obtained by Bruker Avance DMX 400 spectrophotometer with the deuterated solvent as the lock and the residual solvent or TMS as the internal reference.

S2. Synthesis of pillar[5]arene[1,2]

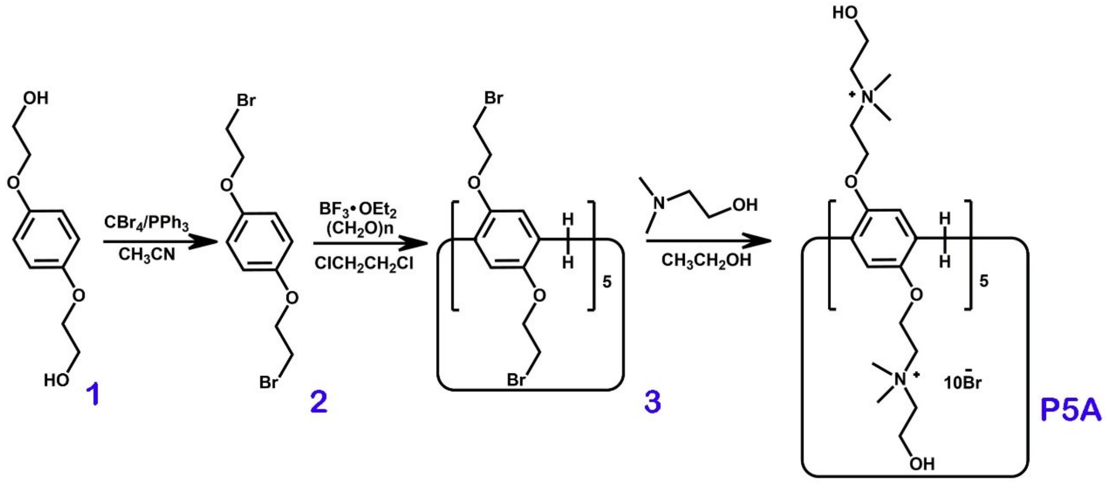

Scheme S1. Synthetic route of pillar[5]arene.

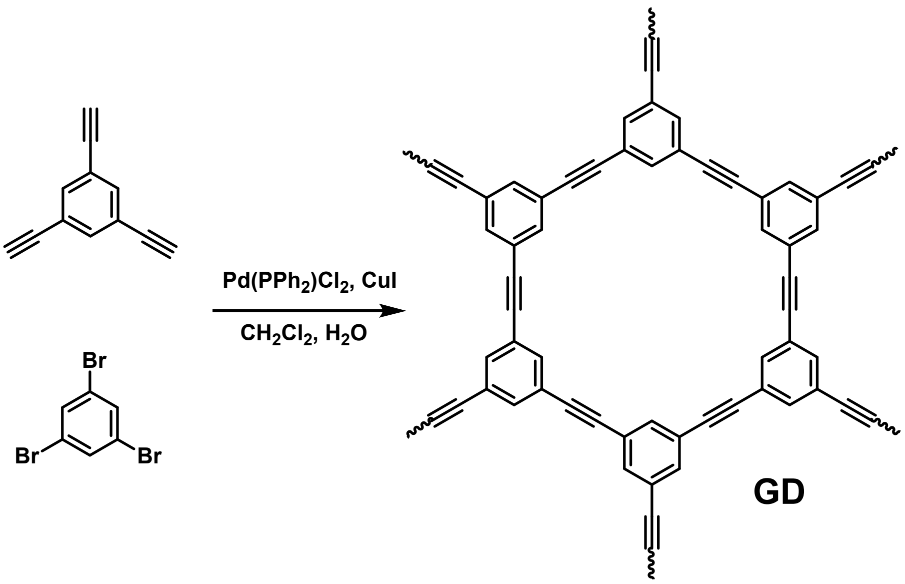

Scheme S2. Synthetic route of GD.

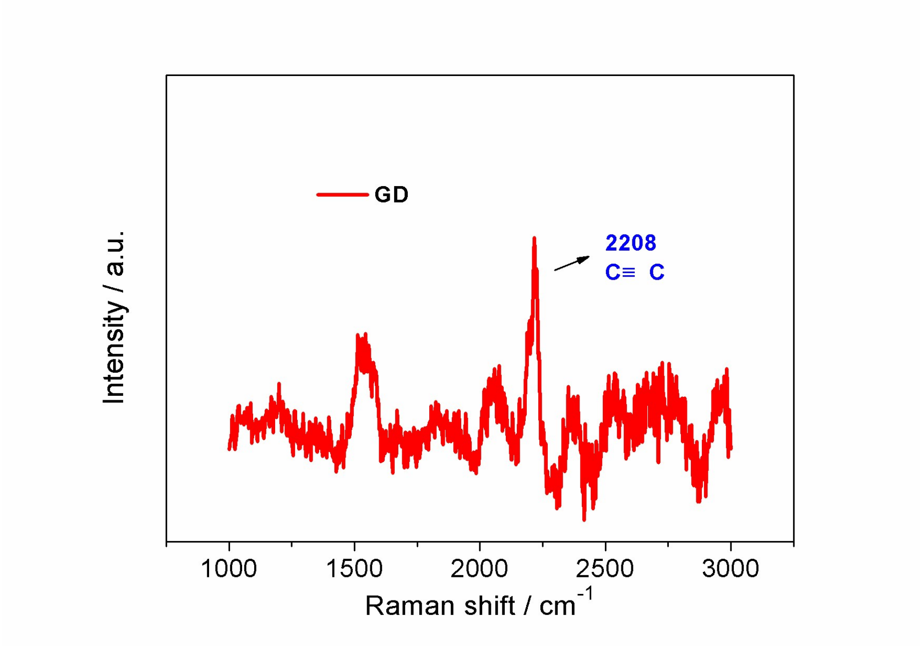

Figure S1. Raman spectrum of GD film.

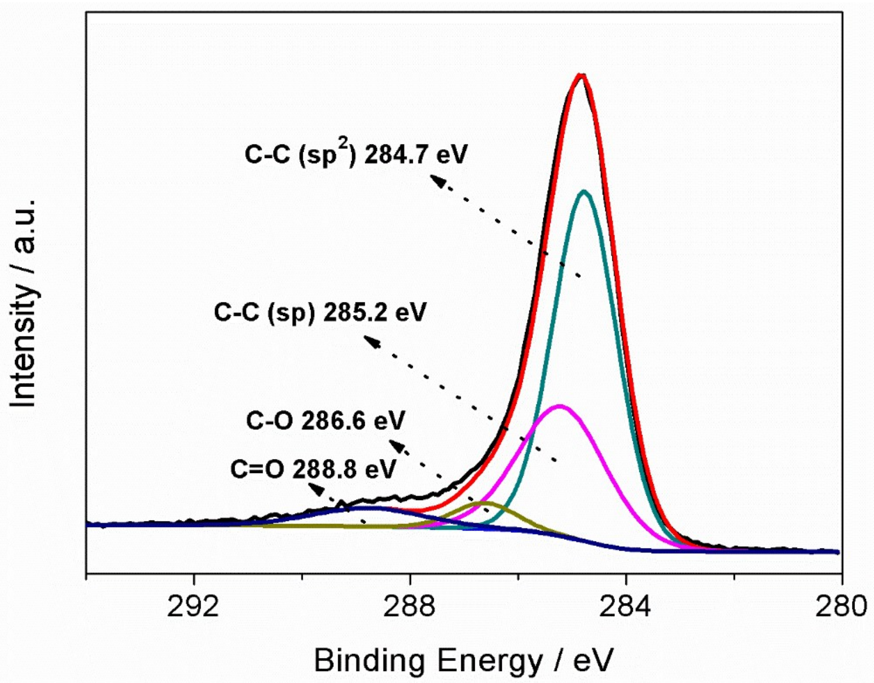

Figure S2. High resolution XPS spectrum of C 1s for GD.

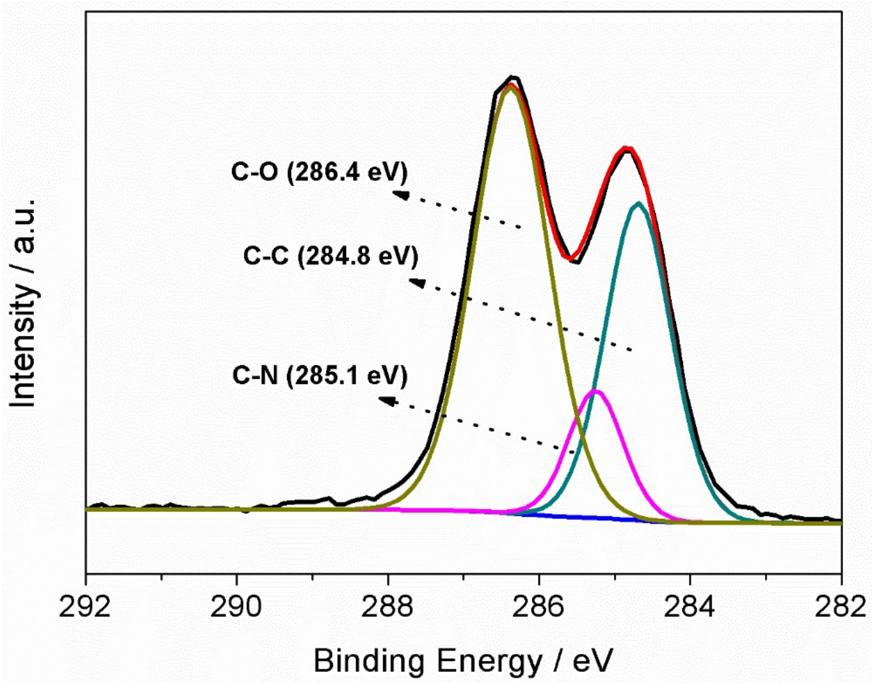

Figure S3. High resolution XPS spectrum of C 1s for P5A.

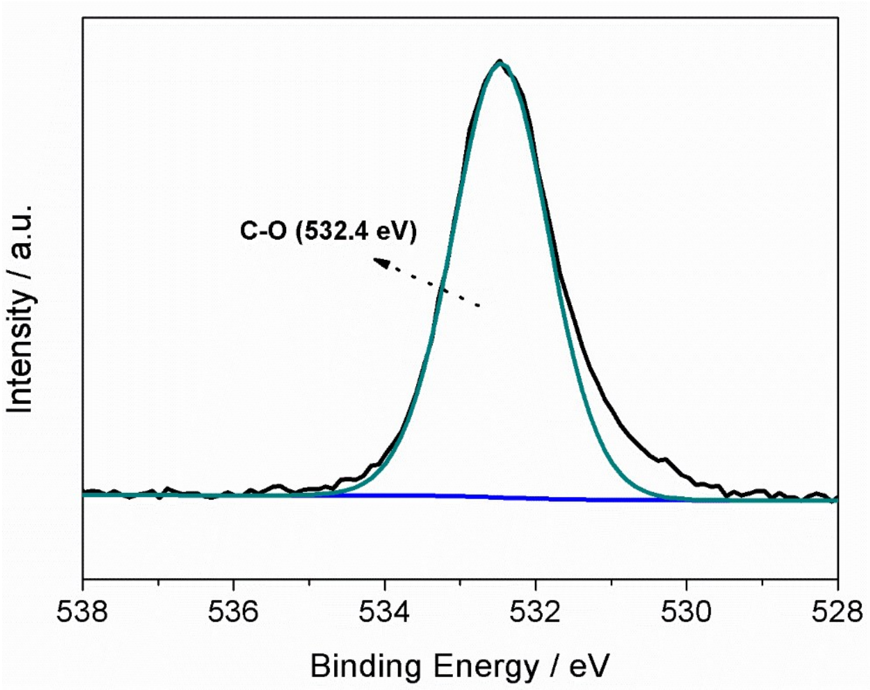

Figure S4. High resolution XPS spectrum of O 1s for P5A.

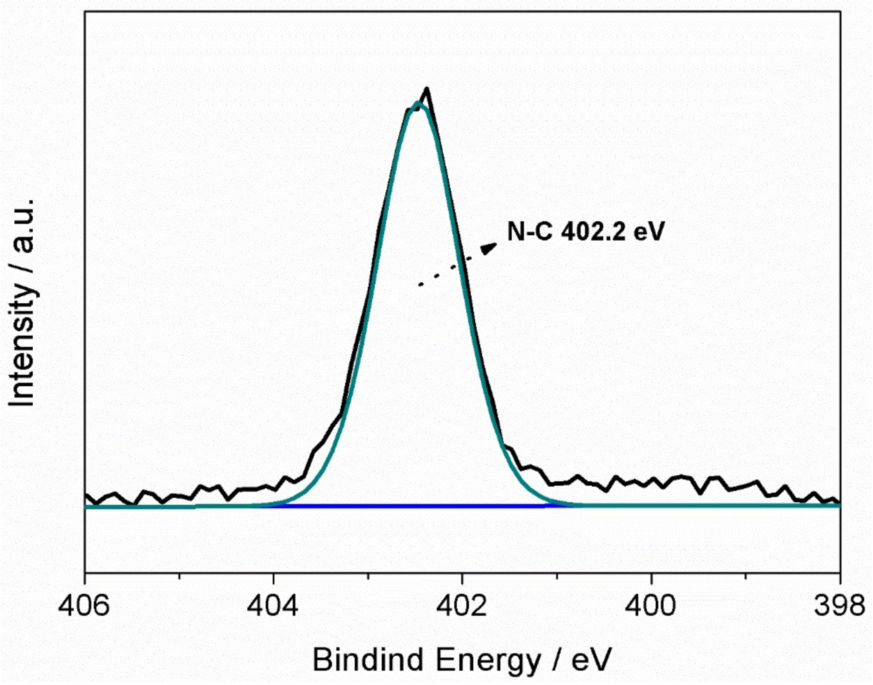

Figure S5. High resolution XPS spectrum of N 1s for P5A.

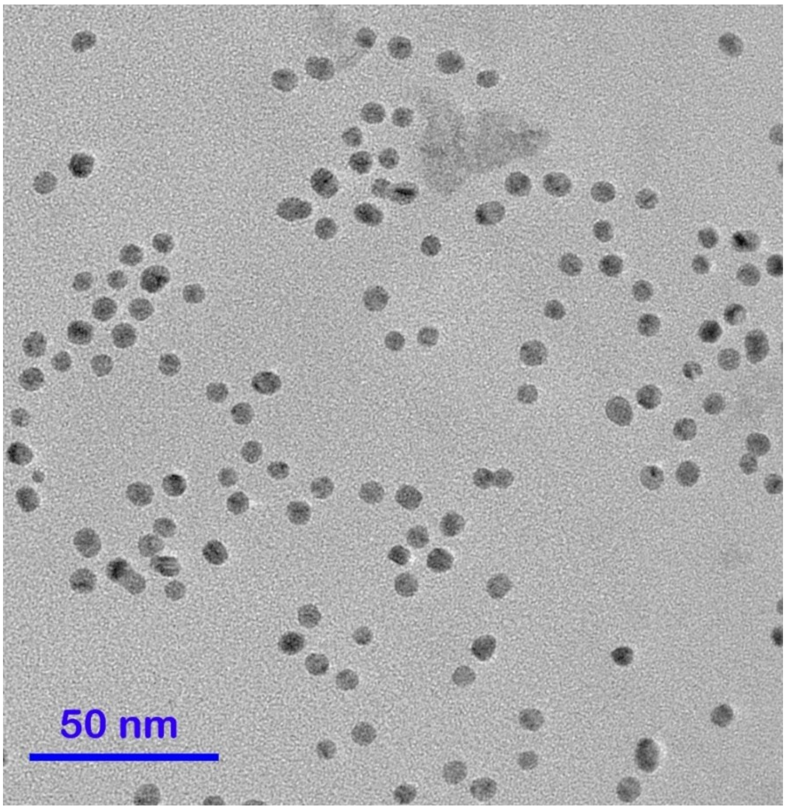

Figure S6. TEM image of P5A modified reduced and stabilized Au nanoparticles.

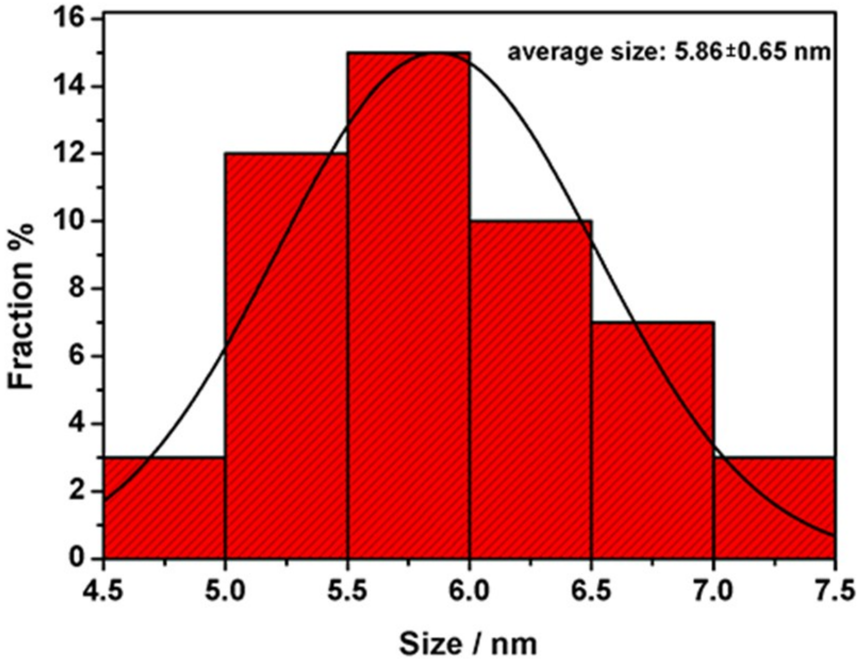

Figure S7. Size distribution of P5A-Au nanoparticles.

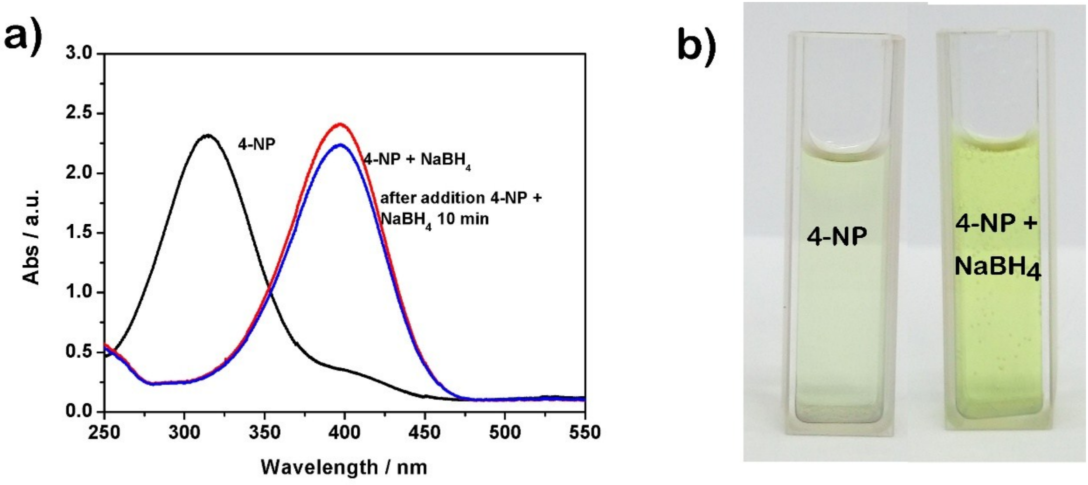

Figure S8. UV-vis spectra of 4-NP before and after adding $NaBH_4$ solution; b) photographs of 4-NP and after adding $NaBH_4$, respectively.

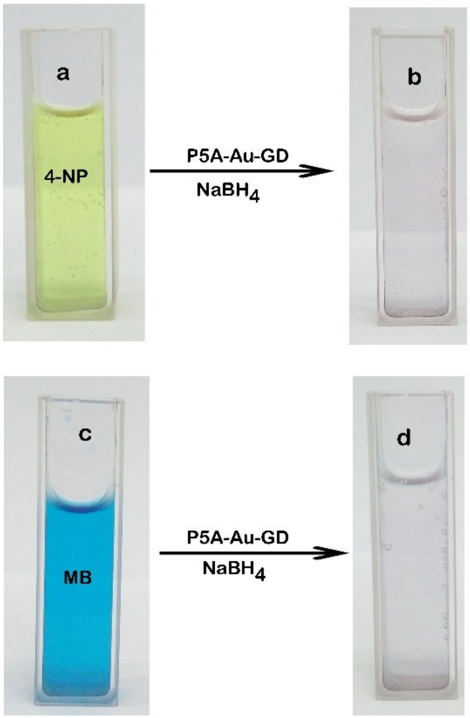

Figure S9. The photographs of aqueous solution of 4-NP reduction before (a) and after (b) in P5A-Au-GD catalyst; the MB degradation before (c) and after (d) in P5A-Au-GD catalyst, respectively.

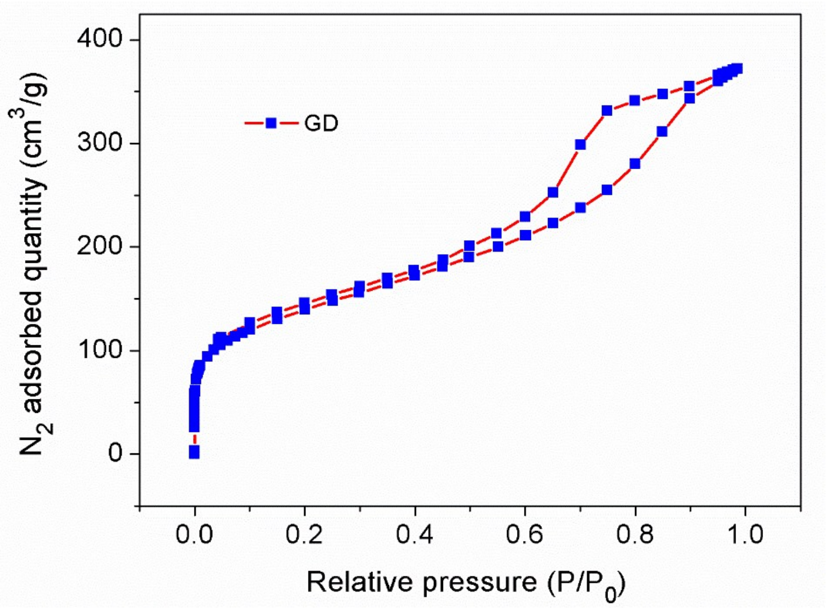

Figure S10. Nitrogen adsorption–desorption isotherm of GD. The porous structure of GD had been carefully studied by the nitrogen adsorption−desorption operation at 77 K. The Brunauer−Emmett−Teller (BET) model reveals that GD has apparent surface areas with 501.5 m^2/g and excellent porous structure.

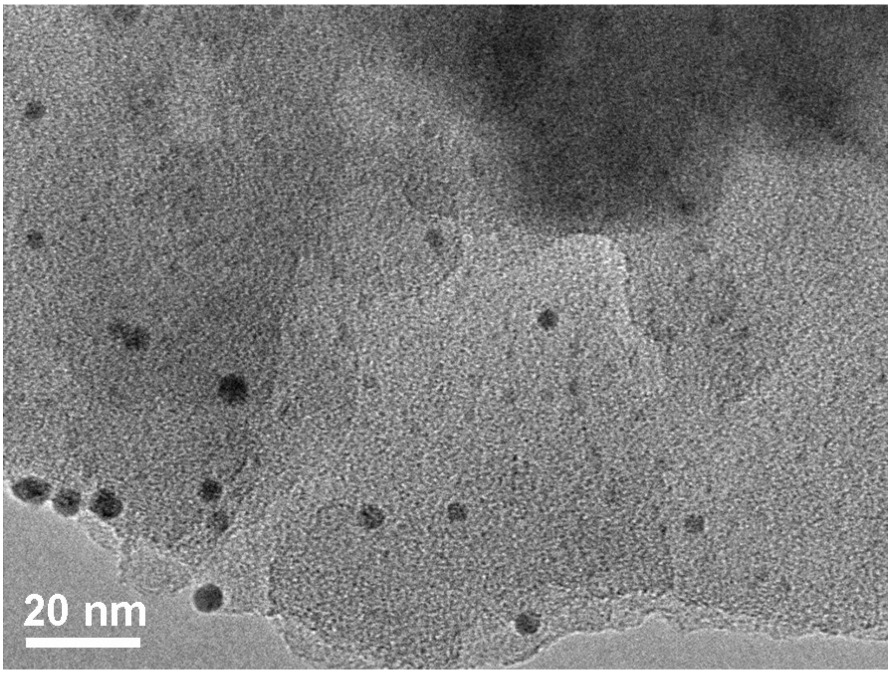

Figure S11. TEM image of used P5A-Au-GD, there is no agminated Au nanoparticles, and the size of Au nanoparticles slightly decrease, implying high stability of P5A-Au-GD catalyst.

Table S1. XPS elemental analysis results (wt. %).

elements	C	O	N	Au
content	64.88	26.46	4.99	3.67

References

1 Y. J. Ma, X. F. Ji, F. Xiang, X. D. Chi, C. Y. Han, J. M. He, Z. Abliz, W. X. Chen and F. H. Huang, *Chem. Commun*., 2011, **47**, 12340–12342.

2 R. Joseph, A. Naugolny, M. Feldman, I. M. Herzog, M. Fridman and Y. Cohen, *J. Am. Chem. Soc*., 2016, **138**, 754–757.
